# Lifestyle and Perceived Well-Being in Children and Teens: Importance of Exercise and Sedentary Behavior

**DOI:** 10.3390/nu17142370

**Published:** 2025-07-19

**Authors:** Nadia Solaro, Gianluigi Oggionni, Giuseppina Bernardelli, Mara Malacarne, Eleonora Pagani, Mariacarla Ferrari, Gianfranco Parati, Daniela Lucini

**Affiliations:** 1 Department of Statistics and Quantitative Methods, University of Milano-Bicocca, 20126 Milan, Italy; nadia.solaro@unimib.it; 2 Exercise Medicine Unit, IRCCS Istituto Auxologico Italiano, 20135 Milan, Italy; g.oggionni@auxologico.it (G.O.); g.bernardelli@unimi.it (G.B.); 3 DISCCO Department, University of Milan, 20122 Milan, Italy; 4 BIOMETRA Department, University of Milan, 20129 Milan, Italy; mara.malacarne@unimi.it; 5 Department of Psychology, Catholic University of the Sacred Heart, 20123 Milan, Italy; eleonora.pagani@unicatt.it; 6 Istituto Leone XIII, 20145 Milan, Italy; mariacarla.ferrari@leonexiii.it; 7 Department of Cardiology, IRCCS Istituto Auxologico Italiano, 20149 Milan, Italy; parati@auxologico.it; 8 School of Medicine and Surgery, University of Milano-Bicocca, 20126 Milan, Italy

**Keywords:** academic performance, behavior, bootstrap, health perception, nonlinear principal component analysis, nonparametric statistics, nutrition, physical activity, prevention, sleep

## Abstract

**Background/Objectives**: Childhood/youth are ideally the best periods to teach healthy behaviors; unfortunately, children/adolescents are frequently characterized by unhealthy lifestyles and reduced well-being. Lifestyle improvement early in life may play a fundamental role in determining present health, preventing many chronic diseases, and fostering well-being. Having a clear picture of the lifestyle characteristics of a group can help institutions and schools define effective educational and intervention strategies. This observational study aims to examine whether information collected from children and adolescents about their lifestyles and perceptions of well-being could help identify particular groups that deserve specific interventions, implemented by the school, to improve their overall health. **Methods**: After administering a simple lifestyle/well-being questionnaire to 225 children/adolescents at an Italian school complex, we investigated the relationships between lifestyles and perceptions of well-being by constructing statistical indicators through nonlinear principal component analysis. Then, we defined lifestyle typologies based on lifestyle indicators and studied the distribution of the well-being indicator across such typologies, also adjusting for sex and age effects. **Results**: The study shows that lifestyle worsens with age (*p* < 0.001) and influences overall well-being perception. We identified four lifestyle typologies by combining two indicators of sedentary behavior and sleep, and of quality of nutrition and the volume of physical activity. The healthier the lifestyle, the better the overall well-being perception is (represented by the indicator that includes the perceived quality of health, sleep, and academic performance) (*p* = 0.005). **Conclusions**: Tailored educational/intervention strategies that consider specific groups’ characteristics, rather than general counseling, might be more effective at improving health/well-being.

## 1. Introduction

The prevention of Chronic Noncommunicable Diseases (CNCDs) represents one of the main goals of health campaigns in clinical practice. In such a context, evidence is available that childhood and youth are ideally the best periods to teach healthy behaviors [1]. Unfortunately, poor nutrition, poor sleep, sedentary behavior, physical inactivity, smoking, and stress, which frequently cluster also in the early stages of life [2], are more and more present at a young age [3], paving the way toward CNCD in adulthood [4] and reduction in well-being [3,5,6]. These observations have suggested an important role of lifestyle changes early in life in determining present health status and the future development of CNCD. Well-being is a complex, multidimensional construct that refers to various factors, including physical/psychological health, a sense of fulfillment, positive functioning, and social components [6]. Nowadays, increasing attention is devoted to well-being in the early stages of life, underlying the critical role of this period in determining health and social roles in adulthood. Children and adolescents may face many pressures and challenges [3], and a reduction in well-being during childhood and youth is associated with stress and anxiety in adulthood. At the same time, at those ages, an individual acquires the physical, emotional, economic, social, and cognitive resources representing the foundation for well-being and good health later in life [3].

In this context, physical exercise has a critical impact on well-being and health, being also capable of leading to broader changes in other health behaviors. International guidelines [7] clearly state that children and adolescents should engage in at least an average of 60 min per day of moderate- to vigorous-intensity, mostly aerobic, physical activity across the week, also incorporating activities that strengthen muscle and bone at least 3 days a week, to foster present and future health. Simultaneously, guidelines recommend limiting sedentary time [7], underlying that sedentary behavior (defined as any waking behavior characterized by an energy expenditure ≤1.5 of Metabolic Equivalents (METs) while in a sitting, reclining, or lying posture [8]) is not synonymous with physical inactivity (defined as an insufficient physical activity level to meet physical activity recommendations) [9]. Although exercise may attenuate the detrimental effects of sedentary behavior, sedentary behavior may reduce the beneficial effects of exercise [10]. Adopting a healthy lifestyle at a young age not only represents the best way to prevent many diseases that may appear in adulthood, but it is also indicated as an essential tool to foster health and well-being in the present. Indeed, more than the fear of developing CNCD later in life, this last consideration may be an efficacious motivational factor in promoting a proactive role for children/teens, as well as their parents, to favor/adopt a healthy lifestyle [11]. Notably, other traditional psychological constructs, known to be predictors of health behavior change (such as outcome expectancies, response efficacy, or attitudes [12]) and more recent, contemporary models (which underline the importance of defining factors capable of moderating the intervention’s effectiveness [13]) have a role in the behavioral change process.

Policies and institutional campaigns to promote a healthy lifestyle must be tailored to individual or group characteristics. To be efficacious, they should focus on specific indications and targets, rather than providing general counseling [14]. Having a clear picture of a group’s lifestyle characteristics may help define educational and intervention strategies aimed at improving health and well-being. In this specific research, we assumed that the routine clinical assessment performed to release clearance for noncompetitive sports participation could also be a convenient opportunity to collect lifestyle and well-being data useful for defining tailored strategies to improve health.

With such a background, this observational study aimed to examine whether the information collected by a simple lifestyle questionnaire, filled in by children/adolescents with the assistance of their parents on the occasion of a routine medical assessment organized by their school, might help individualize particular groups that deserve specific educational and interventional strategies, implemented by the school, to improve their overall health. Using these collected data, we aimed to support our hypothesis that, independently of sex and age, healthy lifestyles are determining factors in improving children’s and teens’ perception of well-being. We inspected our hypothesis using statistical indicators derived from nonlinear principal component analysis [15] and adjusted for sex and age effects. We further hypothesized that certain lifestyles might cluster together, defining distinct indicators that can influence perceptions of well-being. The observed relationships between lifestyles and well-being perception might offer an opportunity to provide evidence for improving lifestyles that could be considered of value by children/teens and their parents, with consequent changes in their lifestyle and betterment of their well-being.

## 2. Materials and Methods

This observational study included data from 352 children/teens (mean age 9.73 ± 2.61 years, range 5–14 years) who were evaluated as required by Italian law to obtain clearance for noncompetitive sports participation. The protocol was approved by the local Institutional Ethics Committee (IRCCS Istituto Auxologico Italiano, Code: 2023_04_18_14, 4 May 2023) and was conducted following the Helsinki Declaration of 1975, as revised in 2008. The assessment took place in an ad hoc room located within a school complex comprising primary and secondary schools. All participants were assessed in the morning hours (08:30–12:30); they were not fasting and were wearing comfortable clothing (tracksuits).

Data were then managed at the Exercise Medicine Unit, IRCCS Istituto Auxologico Italiano. As Italian law requires, the responsible guardians of all children/teens gave written consent to assess them within this campaign frame. The responsible guardians of 253 children/teens (mean age 9.71 ± 2.62 years, range 5–14 years) also gave written consent to use the collected data for scientific purposes in an anonymized and aggregated form.

### 2.1. Clinical Assessment

According to good clinical practice, every child/teen underwent a cardiac, pulmonary, musculoskeletal, and abdominal assessment. A basal electrocardiogram (Cardiofax M, Nihon Kohden, Tokyo, Japan) was performed to verify the absence of evident cardiac diseases that might contraindicate or limit exercise training, particularly at high intensities. All the included individuals were free from any overt disease, as determined by medical assessment and medical history, as well as reports from children/teens and their parents. All electrocardiograms presented sinus rhythm, and all ECG-derived parameters (heart rate, PR interval, QRS complex duration, QT interval, etc.) were within normal limits. All participating individuals received clearance for sports activity.

In all children/teens, weight, height, and Body Mass Index (BMI) were assessed. Weight was measured on participants wearing light clothing without shoes. BMI was calculated as body weight (kilograms) divided by height (meters squared), and BMI percentile was calculated using the CDC—BMI Percentile Calculator for Child and Teen [16].

We took 2–3 measurements of systolic (SAP) and diastolic arterial pressure (DAP) in the supine position using a manual sphygmomanometer (Certus, Tema, Milan, Italy) with an appropriately sized cuff on the right arm after 5 min of rest. We determined the Blood Pressure Percentile for each individual following recent guidelines [17].

### 2.2. Lifestyle Assessment

An ad hoc questionnaire was employed to quantify lifestyle [18]:

- Nutrition was assessed using the American Heart Association (AHA) Healthy Diet Score [19], adapted to Italian eating habits [18], considering fruit/vegetables, fish, sweetened beverages, whole grains, and sodium consumption. The AHA score assumes integer values from 0 (“worst quality”) to 5 (“best quality”).
- Physical activity (total activity volume) was assessed by a modified version of the commonly employed short version of the International Physical Activity Questionnaire (IPAQ) [20,21], which focuses on intensity (nominally estimated in Metabolic Equivalents (METs) according to the type of activity) and duration (in minutes) of physical activity. We decided to employ this questionnaire (as in [20]), even if designed for adults, because it has the advantage of providing a numeric parameter of exercise volume (expressed in METs) that can reflect the total exercise volume. This study considered the following levels: activities of moderate intensity (≈4.0 METs/minute) and activities of vigorous intensity (≈8.0 METs/minute). These levels were used to calculate the total weekly exercise volume of structured exercise using the formula:
  METsMV = 4 × *M* × *dM* + 8 × *V* × *dV*, (1)
  where METsMV stands for moderate and vigorous (MV) physical activity volume expressed in METs minutes/week; *M* is the number of minutes/day of moderate-intensity activities performed in a number *dM* of days/week; *V* is the number of minutes/day of vigorous-intensity activities performed in a number *dV* of days/week.
- The lifestyle questionnaire also inquired about the hours of sleep per night, the hours of sedentary behavior per week (considering the time spent in activities such as studying, reading, screen media use, and transportation), and the individual perception of the quality of sleep, health, and academic performance. These latter were assessed using ordinal evaluation scales ranging from 0 (“worst quality”) to 10 (“best quality”).

This questionnaire was employed in many published studies concerning adults and children/teens [18,20,22]. We decided to limit the lifestyle assessment to the above-described key aspects to balance the need to have an exhaustive picture of individual lifestyle and the need to limit the number of questions. This condition is important to ensure the reliability of the questionnaire, especially if proposed to children and their parents in a clinical setting, and to avoid possible biases in the responses.

All participants voluntarily inserted data with the assistance of their responsible guardians. Appendix A Table A1 summarizes the variables studied and used for statistical analysis.

### 2.3. Statistical Analysis

Preliminarily, we thoroughly inspected the information collected on the 253 children and teens whose responsible guardians provided written consent to participate in scientific studies. As a result of this data quality analysis, participants with incomplete information (24 in all) and those with outlier data because of incongruent responses (4 in all) on the variables listed in Appendix A Table A1 were identified and excluded from the study to prevent any potential biases in the statistical analyses. The study set thus consisted of *n* = 225 participants (mean age 10.24 ± 2.60 years, range 5–14 years).

After that, we carried on with the advanced part of the statistical analyses by relying on a combination of nonparametric and multivariate statistical methods, as they do not require any assumptions about the data, e.g., the normal distribution of variables. We distinguished four analysis steps focused on:

1. Providing preliminary descriptive statistics and nonparametric significance tests [23]. Preliminarily, the sex-by-age-classes distribution of the 225 participants was inspected by testing the equality of age distribution between females and males (or, equivalently, the equality of sex distribution across age classes) through the Chi-square test (with a Monte Carlo (MC) procedure to obtain the *p*-value). Next, descriptive statistics of anthropometric, hemodynamic, and lifestyle variables and perceived quality scales were computed as median ± MAD (Median Absolute Deviation) both within sex and age classes and over all the participants. Then, we performed the two-tailed Kruskal–Wallis and median MC tests for each variable to evaluate the null hypothesis of the absence of differences between males and females and across age classes. Besides this, we constructed the correlogram of the ten variables we selected for their relevance to our investigation, i.e., sex (used as a stratification variable), age, BMI, lifestyle variables, and perceived quality scales (Appendix A Table A1). Spearman’s rank correlation coefficients were reported for every pair of variables on the upper triangular part of the correlogram, along with significance test results for uncorrelation concerning the whole set of 225 participants and the distinction between females and males. Moreover, on the correlogram diagonal, graphs displaying the univariate within-sex distributions were built for each variable, while in the lower triangular part, bivariate plots displaying the joint distributions of each pair of variables were built consistently with their measurement level.
2. Setting up lifestyle indicators. We applied the nonlinear (or categorical) principal component analysis method, i.e., a nonlinear multivariate data analysis technique for dimensionality reduction, also known as PRINCALS [15], to account for the relationships among the four lifestyle variables, i.e., sedentary time, sleep hours, physical activity volume, and nutrition quality, and obtain a few standardized and uncorrelated dimensions, which we regarded as lifestyle statistical indicators. Unlike the standard principal component analysis, PRINCALS allows variables with different measurement levels to be jointly handled in a unique analysis through an optimal scaling procedure (Appendix A Table A1), through which qualitative variables are iteratively quantified during the extraction process of dimensions [15]. Component loadings (i.e., the correlation coefficients between the optimally scaled variables and the obtained PRINCALS dimensions) were rotated through the varimax method to simplify the interpretation of the dimensions. We used a threshold of 0.6 in absolute value on component loadings to identify the variables that more strongly contributed to the dimension construction and interpretation. We kept in analysis a number *q* of the first dimensions such that each explained at least 20% of the total variance of the optimally scaled variables and together accounted for a cumulative percentage of this variance of not less than 60%. To be used as lifestyle indicators, the retained dimensions were then checked to assume scores in a direction consistent with the meaning of the original lifestyle variables. Specifically, because the dimensions are zero-mean by construction, higher and positive dimension scores were supposed to represent children/teens with the healthiest lifestyles, while lower and negative scores had to indicate children/teens with the least healthy lifestyles.
  Lastly, we assessed the stability of the extracted dimensions by applying the nonparametric stratified balanced bootstrap [24]. In practice, *B* = 5000 bootstrap samples were generated at random so that each subject appeared exactly *B* times over the whole set of the *nB* = 225(5000) = 1,125,000 replicates (balanced bootstrap), and the same percentages of sex-by-age classes were reproduced in each sample (stratified bootstrap). The first *q* dimensions were extracted on each bootstrap sample with the component loadings rotated through the varimax method. Then, Procrustes rotations were employed to overcome the problem of configurations not aligned with the PRINCALS solution in the original dataset [25]. The stability of the indicators was subsequently assessed by computing 90% bootstrap confidence intervals (CIs) for the “Variance Accounted For” (VAF) measure and the component loadings using the bias-corrected and accelerated (BC_a_) method and the option “infinitesimal jackknife” [24].
3. Defining lifestyle typologies based on the obtained lifestyle indicators. We set up lifestyle typologies of children and teens by combining their negative or nonnegative (i.e., positive or equal to zero) scores on the lifestyle indicators. Nonnegative scores on all indicators characterized the healthiest lifestyle typology, while negative scores denoted the least healthy lifestyle typology; additionally, indicator scores with discordant signs defined intermediate typologies. Once the typologies were defined, we analyzed the sex and age classes composition of each of them to disclose any possible differences. In particular, we tested the equality of sex and age-class distributions across the typologies through the Chi-square MC test. Cramer’s V, along with 95% bootstrap CIs (built with the BC_a_ method based on *B* = 1000 bootstrap samples), was also computed to provide a measure of the strength of association between lifestyle typologies and sex, and lifestyle typologies and age classes, respectively.
4. Setting up perceived quality and BMI indicators and inspecting their distributions within the lifestyle typologies. Similarly to the construction of lifestyle indicators, we applied the PRINCALS method to account for the interrelationships among the three ordinal perceived quality scales and BMI (Appendix A Table A1) and set up standardized and uncorrelated dimensions. We applied the same approach described before for the lifestyle indicators to rotate the component loadings, choose the ideal number of dimensions to keep in analysis, assess the direction of dimension scores, and evaluate the stability of the VAF measure and component loadings through 90% bootstrap CIs. After that, to investigate whether different lifestyles affect perceived quality and BMI, we first depicted the score distributions of these indicators within the lifestyle typologies using violin plots (with box plots on their inside). Then, we tested the overall null hypothesis of no typology effects on them using the two-tailed Kruskal–Wallis and median MC tests, as well as the one-tailed Jonckheere–Terpstra permutation test, this latter having as the alternative hypothesis the progressive bettering of perceived quality and BMI with healthier lifestyles. In rejecting the overall null hypothesis, we deepened the analysis by comparing the distribution of the same indicator between every two typologies with the one-tailed Kolmogorov–Smirnov MC test and the Jonckheere–Terpstra permutation test. To preserve the nominal significance level of the overall null hypothesis, in these pairwise comparisons, we adjusted the *p*-values using the False Discovery Rate (FDR) method [26].

After that, to study the distributions of these indicators within the lifestyle typologies, controlling for sex and age, we conducted the same analysis described above using sex- and age-adjusted indicators of perceived quality and BMI. To adjust for sex and age effects, we first fitted a general linear model including each indicator *Y* as a dependent variable and sex, age, and their interaction as predictors according to the equation:

(2) $$y_{i}=\mu +\alpha \;{sex}_{i}+\beta {\;age}_{i}+\gamma {\;sex}_{i}\;{age}_{i}+e_{i}$$

where $y_{i}$ is the score of the *i*-th subject on *Y*; ${sex}_{i}$ is equal to 0 for females and 1 for males; ${\;age}_{i}$ is the age of the *i*-th subject; *μ* is the constant of the model (indicating the mean indicator score of females at age 0); *α* is the sex effect; *β* is the age effect; *γ* is the sex-by-age interaction effect; $e_{i}$ is the *i*-th model residual (*i* = 1, …, 225). The model residuals ${\hat{e}}_{i}$, computed as differences between the original indicator scores $y_{i}$ and the model predicted scores ${\hat{y}}_{i}$:${\hat{e}}_{i}=y_{i}-{\hat{y}}_{i}$, for each *i* = 1, …, 225, were regarded as scores of adjusted indicators since they are free of sex and age effects by construction.

Finally, to estimate the effect sizes of the lifestyle typology memberships on the obtained perceived quality and BMI indicators, we fitted a series of quantile regression models [27] to explain the relationships between the three quartiles τ of the indicator distributions (or, also, the 25th, 50th, and 75th percentiles) and the lifestyle typology memberships, also controlling for sex and age effects. Formally, to estimate the effect sizes of the lifestyle typologies without controlling for sex and age, we built the quantile regressions with the least healthy lifestyle typology as the reference category (denoted by $t_{1}$) according to the equations:

(3) $$Q^{\left(\tau \right)}\left(y_{i}|t_{gi}\right)=\eta_{0}^{\left(\tau \right)}+\sum_{g=2}^{G}\eta_{g}^{\left(\tau \right)}t_{gi}$$

where τ=0.25, 0.5, 0.75 denotes the specific indicator quantile; $t_{gi}$ is equal to 1 if the *i*-th subject belongs to the lifestyle typology g, and 0 otherwise (g=2, …, G); $\eta_{0}^{\left(\tau \right)}$ is the constant of the model (indicating the τ-th indicator quantile in the reference typology $t_{1}$); $\eta_{g}^{\left(\tau \right)}$ is the quantile regression coefficient for the lifestyle typology g, which provides a direct evaluation of its effect size on the τ-th indicator quantile, expressing it as a difference with respect to the reference typology $t_{1}$.

Moreover, to estimate the effect sizes of the lifestyle typologies, controlling for sex and age effects, we built the following quantile regressions parameterized with respect to the reference typology $t_{1}$ based on the equations:

(4) $$Q^{\left(\tau \right)}\left(y_{i}|t_{gi},{sex}_{i},{age}_{i}\;\right)=\theta_{0}^{\left(\tau \right)}+\sum_{g=2}^{G}\theta_{g}^{\left(\tau \right)}t_{gi}+\alpha^{\left(\tau \right)}\;{sex}_{i}+\beta^{\left(\tau \right)}{\;age}_{i}+\gamma^{\left(\tau \right)}{\;sex}_{i}\;{age}_{i}$$

for τ=0.25, 0.5, 0.75 and g=2, …, G, where $\theta_{0}^{\left(\tau \right)}$ is the constant of the model (indicating the τ-th indicator quantile for females at age 0 in the reference typology $t_{1}$); $\theta_{g}^{\left(\tau \right)}$ expresses the effect size of the lifestyle typology g on the τ-th indicator quantile free of sex and age effects; the parameters $\alpha^{\left(\tau \right)}$, $\beta^{\left(\tau \right)}$, and $\gamma^{\left(\tau \right)}$ preserve the same meaning as Equation (2) for a fixed τ, but are free of lifestyle typology effects.

Quantile regression results will be presented in the form of standardized parameter estimates (to detect, for a fixed τ, the effect with the highest magnitude in absolute value), 95% bootstrap CIs, and *t*-tests with standard errors computed using *B* = 1000 bootstrap samples [27]. The goodness-of-fit index $R^{1}(\tau )$, defined in [28] as an analog of the $R^{2}$ index for Ordinary Least-Squares (OLS) regression models, will also be provided. However, although $R^{1}(\tau )$ is normalized in the [0, 1] interval, it should be regarded more appropriately as a pseudo-$R^{2}$ index for quantile regression and, as such, cannot be interpreted directly as the proportion of variance explained by the model, since $R^{1}$ indicates the reduction in the objective function of quantile regression compared to a model that includes only the intercept (i.e., the null model) [28]. In this sense, even small increases in $R^{1}$ can be meaningful when compared to the null model. As an expected behavior in practice, its values are typically low and, in general, lower than the $R^{2}$ values of OLS regressions, reflecting the nature of the objective function of quantile regression [27,28]. Therefore, in this context, the $R^{1}$ values will be mainly used to assess how predictors affect different parts (represented by the quartiles) of the indicator distributions and, in particular, to see whether there exist sensible differences as moving from the unadjusted quantile regressions in (3) to the adjusted quantile regressions in (4).

Throughout the study, the nominal test significance level was set at 0.05. We performed the statistical analyses using the R software, version 4.4.2 [29], with the following contributed packages: “boot” [30] for the bootstrap; “coin” [31] for the MC version of the Chi-square, Kruskal–Wallis, and median tests; “DescTools” [32] for the Jonckheere–Terpstra permutation test; “Gifi” for PRINCALS [33]; “GGally” for the correlogram [34]; “ggplot2” [35] for the construction of the other graphs, “quantreg” for the quantile regressions [36] and “jtools” for the construction of bootstrap CIs of model parameters and computation of the $R^{1}$ index [37]; “rcompanion” for the computation of Cramer’s V and its bootstrap CIs [38].

## 3. Results

### 3.1. Preliminary Descriptive Statistics and Nonparametric Significance Tests

In the study set, 107 are females (47.6% out of 225) and 118 males (52.4%), distributed similarly over the age classes (mean age 10.24 ± 2.60 years, range 5–14 years). No significant difference is detected by the Chi-square test in the subjects’ distribution by sex and age classes (Appendix A Table A2).

Table 1 and Table 2 display descriptive statistics (median ± MAD) of the variables under study and significance tests computed by splitting the children and teens into sex and age classes, respectively. Overall, females and males show significant differences on both tests only regarding waist circumference, SAP, and METs of moderate and vigorous physical activity (METsMV) (Table 1). Interestingly, males have a higher METsMV median than females (2120 ± 760 vs. 1680 ± 480). Conversely, no significant differences between females and males in the other lifestyle variables or the perceived quality scales are detected.

Comparison analysis across age classes (Table 2) produces more significant results on both tests. All the anthropometric variables show significant differences except BMI percentiles, as expected. However, it is worth noting that the median values of BMI not expressed in population percentiles progressively increase with age classes from the youngest children (15.32 ± 0.86) to teens (18.77 ± 1.90). In addition, SAP and DAP percentiles appear to differ among age classes, being lower in teens, though in the normal range [39].

Regarding the other variables, both tests detect significant differences in sedentary time and sleep hours, progressively worsening as age increases. A similar trend (though only the Kruskal–Wallis test is significant) is also visible in the AHA score: the youngest children (5–7 years) have a median score of 3 ± 1 compared to 2 ± 1 of the subsequent age classes. At the same time, no significant difference regarding METsMV is found among age classes. Finally, the perceived quality scale of academic performance significantly differs among age classes according to both tests: a progressive impairment is observed from younger children (5–7 and 8–10 years), with a median score of 9 ± 1, to older children (11–12 years) and teens (13–14 years), with a median score of 8 ± 1. A similar trend is visible for the perceived sleep quality scale: children have a median score of 9 ± 1 against teens with 8.5 ± 0.5. In contrast, no significant difference is detected in the perceived health quality scale.

**Figure 1:**
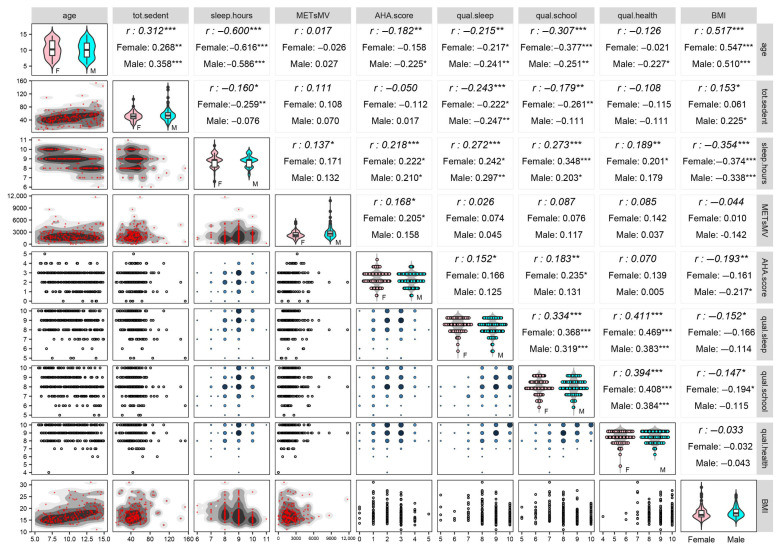
Correlogram of the study variables (Appendix A Table A1) with Spearman’s rank correlation coefficients and significance test for uncorrelation over the whole set of *n* = 225 children and teens and within females and males. Spearman’s rank correlation coefficients *r* (upper triangular part) are computed over the whole subject set and within the female and male groups. Two-tailed exact test for uncorrelation: $H_{0}:\rho \left(X_{j},X_{v}\right)=0$ against $H_{1}:\rho \left(X_{j},X_{v}\right)\neq 0$ for each pair (j,v), j≠v, where $\rho \left(X_{j},X_{v}\right)$ is Spearman’s rank correlation coefficient of variables $X_{j}$ and $X_{v}$ in the population. Significance level: * *p* < 0.05, ** *p* < 0.01, *** *p* < 0.001. Graphs on the diagonal display the within-sex distributions for each variable (F = Female, M = Male). Continuous variables: violin plots with box plots on their inside; outliers are depicted with a black circle. Ordinal variables: beeswarm plots with violin plots in the background. Bivariate plots on the lower triangular part: Two continuous variables: two-dimensional density plots with shaded highest-density point regions. One-ordinal variable-one continuous variable (except sleep hours): scatter plots. Two-ordinal variables: bubble plots with circle sizes proportional to the counts (also used for sleep hours because of its low number of distinct values).

Further analyses concerning the bivariate relationships among age, lifestyle variables, perceived quality scales, and BMI are provided in the correlogram (Figure 1), where Spearman’s rank correlation coefficients *r* (reported in the upper triangular part) are computed over the whole subject set and within females and males.

The correlogram illustrates the complexity of the bivariate relationships among the variables. By the first row of panels, it can be seen that age is negatively correlated with sleep hours (*r* = −0.600, *p* < 0.001) and the AHA score (*r* = −0.182, *p* < 0.01) and positively with sedentary time (*r* = 0.312, *p* < 0.001). In addition, the perceived quality scores, especially of academic performance (*r* = −0.307, *p* < 0.001), tend to reduce with age, while BMI, as expected, increases (*r* = 0.517, *p* < 0.001). Moreover, it is worth noticing that sleep hours correlate negatively with sedentary time (*r* = −0.160, *p* < 0.05), especially in females (*r* = −0.259, *p* < 0.01), and positively with the AHA score (*r* = 0.218, *p* < 0.001) and METsMV (*r* = 0.137, *p* < 0.05). On the other hand (last four columns-rows of panels), a relatively intense positive relation links the three perceived quality scales together, in particular, the perceived sleep and health quality scales (*r* = 0.411, *p* < 0.001), which is stronger in females (*r* = 0.469, *p* < 0.001). In addition, sleep hours correlate positively with the three perceived quality scales (sleep: *r* = 0.272, *p* < 0.001; academic performance: *r* = 0.273, *p* < 0.001; health: *r* = 0.189, *p* < 0.01) and negatively with BMI (*r* = −0.354, *p* < 0.001) (third row with the last four columns); the opposite trend can be seen instead for sedentary time (second row with the last four columns), in particular, its negative relation with the perceived quality scales of sleep (*r* = −0.243, *p* < 0.001) and academic performance (*r* = −0.179, *p* < 0.01). Lastly, the AHA score correlates positively with the perceived quality scales of sleep (*r* = 0.152, *p* < 0.05) and academic performance (*r* = 0.183, *p* < 0.01) and negatively with BMI (*r* = −0.193, *p* < 0.01) (fifth row with the last four columns). Therefore, the correlogram, expressing the intertwining system of interrelationships among variables, is the starting point of PRINCALS, which is used to obtain synthetic representations of such relationships in fewer uncorrelated dimensions.

### 3.2. Lifestyle Indicators

Table 3 reports the rotated component loadings with 90% bootstrap CIs of the four lifestyle variables and the first two PRINCALS dimensions kept in the analysis according to the criteria described in Section 2.3. Such dimensions together explain 61.75% (90% CI: (59.46%, 66.33%)) of the total variance. Dimension 1 accounts for 32.63% (90% CI: (31.43%, 37.69%)) of the total variance, and because it correlates highly and positively with sleep hours and highly and negatively with sedentary time, it represents an overall indicator of sedentary behavior and sleep (acronym SedSlee). Specifically, its higher and positive scores identify children and teens who spend more hours per night of sleep and/or fewer hours per week in sedentary behavior. Conversely, its lower and negative scores identify children and teens with fewer hours/night of sleep and/or more hours/week of sedentary behavior. Dimension 2 reproduces 29.12% (90% CI: (25.53%, 31.34%)) of the total variance. As it correlates highly and positively with the AHA score and METs of moderate and vigorous physical activity, it can be interpreted as an overall indicator of the quality of nutrition and physical activity volume (acronym QunPha). In particular, its higher and positive scores identify children and teens following healthier alimentary habits and/or enjoying more physical activity. Vice versa, its lower and negative scores identify children and teens with less healthy alimentary habits and/or enjoying less physical activity.

### 3.3. Lifestyle Typologies

**Figure 2:**
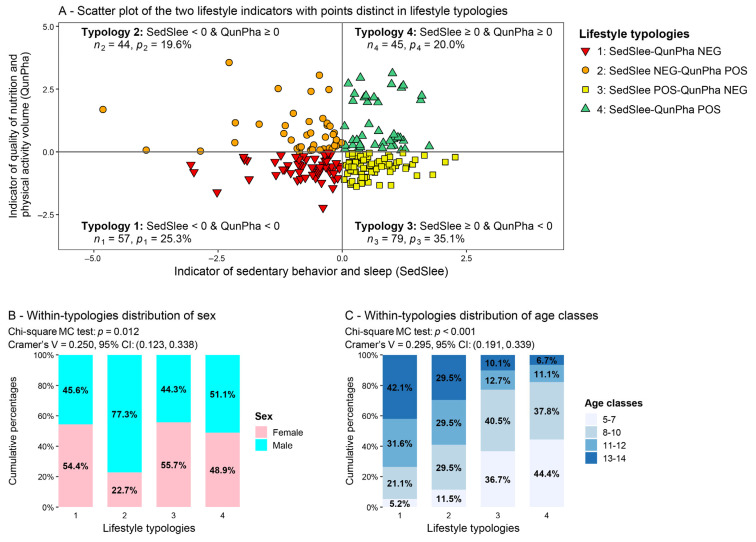
Characterization of the four lifestyle typologies. (**A**) Within-typology frequency of children/teens: $n_{g}$; within-typology percentage: $p_{g}=\frac{n_{g}}{225}100\%$, g=1, 2, 3, 4. (**B**,**C**) Percentages displayed in the bar charts: $\frac{n_{cg}}{n_{g}}100\%$, where $n_{cg}$ is the frequency of children/teens within females and males (**B**) or age classes (**C**) in typology g, with g=1, 2, 3, 4.

By mutually combining negative and nonnegative (i.e., zero or positive) scores of the two lifestyle indicators, we obtained the four lifestyle typologies described and depicted in Figure 2. The scatter plot in Figure 2A refers to the two lifestyle indicators (the SedSlee indicator in the abscissa and the QunPha indicator in the ordinate). It contains points representing the children/teens distinguished by symbols and colors according to the four lifestyle typologies. In particular, given its negative scores on both lifestyle indicators, typology 1 (“SedSlee-QunPha NEG,” red down triangles in the third quarter of the graph) refers to the least healthy lifestyle and comprises 25.3% of children and teens. In contrast, typology 4 (“SedSlee-QunPha POS,” green up triangles in the first quarter), deriving from nonnegative scores on both lifestyle indicators, refers to the healthiest lifestyle and includes 20% of children and teens. The other two typologies are in an intermediate position. Typology 2 (“SedSlee NEG-QunPha POS,” orange circles in the second quarter), with 19.6% of children/teens, is obtained by combining the negative SedSlee indicator scores with the nonnegative QunPha indicator scores. Typology 3 (“SedSlee POS-QunPha NEG,” yellow squares in the fourth quarter), with 35.1% of children/teens, is obtained by combining the nonnegative SedSlee indicator scores with the negative QunPha indicator scores.

To have a fuller picture of the typology composition, Figure 2B,C report 100% stacked bar charts of the within-typologies distributions of sex and age classes, respectively, along with the Chi-square MC test results and Cramer’s V values. In both cases, the distributions of sex and age classes differ significantly across the typologies (sex: *p* = 0.012; age: *p* < 0.001), and Cramer’s V values indicate associations of moderate intensity between the lifestyle typologies and, respectively, sex (Figure 2B, V = 0.250, 95% CI: (0.123, 0.338)) and age classes (Figure 2C, V = 0.295, 95% CI: (0.191, 0.339)). Regarding sex distribution (Figure 2B), a slight prevalence of females in typologies 1 (54.4% out of 57 subjects) and 3 (55.7% out of 79 subjects) can be immediately noted. In comparison, males are remarkably prevalent in typology 2 (77.3% out of 44 subjects) and slightly prevalent in typology 4 (51.1% out of 45 subjects). Interestingly, most females tend to have negative scores on the QunPha indicator, unlike males, who mostly have positive scores. This result is strongly driven by the higher values of moderate and vigorous physical activity METs in males than females, as shown in Table 1 by the median values.

On the other hand, Figure 2C, reports the distribution of age classes within the lifestyle typologies. A progressive reduction in age can be immediately noticed, moving from the least healthy typology 1 through the intermediate typologies 2 and 3 to the healthiest typology 4. In particular, teens (13–14 years) represent 42.1% of subjects in typology 1 (out of 57) and 6.7% in typology 4 (out of 45), in contrast to the youngest children (5–7 years), who represent 5.2% of subjects in typology 1 (out of 57) and 44.4% in typology 4 (out of 45). This result further confirms that sleep hours, sedentary behavior, and alimentary habits worsen as age increases. Since the age composition of the four typologies markedly changes in progression with the age classes, the order of the lifestyle typologies will be considered as such in the subsequent analyses.

### 3.4. Perceived Quality and BMI Indicators and Analysis Within the Lifestyle Typologies

Table 4 contains the rotated component loadings with 90% bootstrap CIs of the three perceived quality scales and BMI with the first two PRINCALS dimensions maintained in the analysis, which together explained 70.62% (90% CI: (68.41%, 75.32%)) of the total variance. Dimension 1, which reproduces 46.82% (90% CI: (43.25%, 51.94%)) of the total variance, is highly and positively correlated with all three perceived quality scales and is then interpreted as an indicator of perceived well-being. Children and teens with higher and positive scores on this indicator have a better perceived quality of health, academic performance, and sleep. In contrast, lower and negative scores identify children and teens with opposite characteristics. Dimension 2, which explains 23.79% (90% CI: (22.08%, 26.76%)) of the total variance, correlates highly and positively with BMI only. Accordingly, it can be interpreted as a BMI indicator, which, unlike the original BMI, has the advantage of being uncorrelated with the perceived well-being indicator. High and positive scores on the BMI indicator indicate children/teens with a high BMI, while low and negative scores identify children/teens with a low BMI.

**Figure 3:**
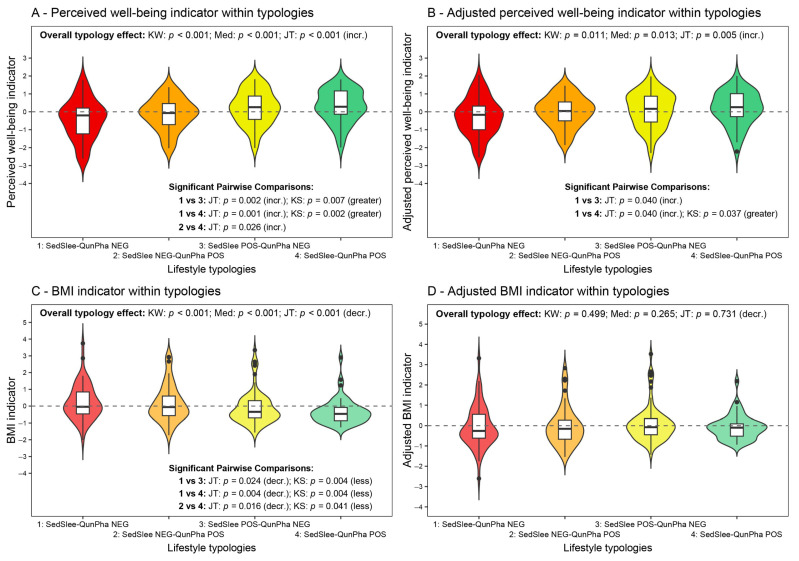
Violin plots of the perceived well-being and BMI indicators (along with sex-by-age adjusted indicators) within the four lifestyle typologies. Outliers are depicted with a black circle. The Kruskal–Wallis (KW), median (Med), and Kolmogorov–Smirnov (KS) Monte Carlo tests are based on 10,000 samples. The Jonckheere–Terpstra (JT) test is based on 5000 permutations. Reported *p*-values concerning significant pairwise comparisons are adjusted with the FDR method (see Section 2.3). The meaning of the two-tailed Kruskal–Wallis and median tests is similar to the one provided below in Table 2. One-tailed Jonckheere–Terpstra test, overall typology effect: $H_{0}:\tau_{1}=\tau_{2}=\tau_{3}=\tau_{4}$ against the increasing alternative $H_{1}:\tau_{1}\leq \tau_{2}\leq \tau_{3}\leq \tau_{4}\;$(incr.; (**A**,**B**)), or the decreasing alternative $H_{1}:\tau_{1}\geq \tau_{2}\geq \tau_{3}\geq \tau_{4}\;$(decr.; (**C**,**D**)), with at least one strict inequality, where $\tau_{g}$ is the effect of the *g*-th typology on indicator *Y*. One-tailed Jonckheere–Terpstra test, pairwise comparisons: $H_{0}:\tau_{g}=\tau_{k}$ against $H_{1}:\tau_{g}<\tau_{k}\;$(incr.; (**A**,**B**)), or $H_{1}:\tau_{g}>\tau_{k}\;$(decr.; (**C**)), for each pair of typologies (g,k), with g<k. One-tailed Kolmogorov–Smirnov test, pairwise comparisons: $H_{0}:F_{g}\left(y\right)=F_{k}(y)$, for all *y*, against $H_{1}:F_{g}(y)<F_{k}(y)$ (greater; (**A**,**B**)), or $H_{1}:F_{g}(y)>F_{k}(y)$ (less; (**C**)), for at least one *y*, and for each pair of typologies (g,k), g<k, where *F* is the cumulative distribution function of indicator *Y*.

After construction, these two indicators were adjusted for sex and age effects (see Section 2.3). Figure 3 displays the violin plots (with box plots on their inside) of the distributions of these indicators in both the nonadjusted and adjusted versions within the four lifestyle typologies. As for the nonadjusted indicators, the perceived well-being indicator (Figure 3A) and the BMI indicator (Figure 3C) differ significantly across the four lifestyle typologies. In particular, by the Jonckheere–Terpstra test, the perceived well-being indicator scores tend to increase significantly from typology 1 (SedSlee-QunPha NEG) to typology 4 (SedSlee-QunPha POS) (*p* < 0.001), while, as expected, the BMI indicator scores decrease significantly (*p* < 0.001).

Concerning the pairwise contrasts performed through the Kolmogorov–Smirnov and Jonckheere–Terpstra tests with the FDR *p*-value adjustment, it is worth noting that the only significant results regard the comparisons “typology 1 vs. 3,” “typology 1 vs. 4” (this latter as expected), and “typology 2 vs. 4” for both indicators. These results suggest that adequate sleep hours with limited sedentary behavior are crucial in improving the individual perception of well-being. The “typology 1 vs. 3” comparison means moving from negative to positive scores on the SedSlee indicator conditionally on negative QunPha indicator scores. Conversely, the “typology 2 vs. 4” comparison regards moving from negative to positive scores on the SedSlee indicator conditionally on positive QunPha indicator scores. Therefore, under the same conditions regarding nutrition quality and physical activity volume, less sedentary time and more sleep hours significantly improve perceived well-being. However, although similar remarks might be advanced for the BMI indicator, the analysis conducted at this level does not consider the possible confounding role of sex and age.

Indeed, after controlling for sex and age effects, the distribution of the perceived well-being indicator (Figure 3B) still significantly differs across the four lifestyle typologies, while the BMI indicator (Figure 3D) does not. In particular, by the Jonckheere–Terpstra test, the adjusted perceived well-being indicator scores tend to increase significantly from typology 1 to typology 4 (*p* = 0.005). At the same time, the unique significant pairwise comparisons remain “typology 1 vs. 4” and “typology 1 vs. 3.” This latter finding suggests that net of sex and age and in the presence of poor nutrition quality and insufficient physical activity, less sedentary time and more sleep hours still tend to contribute to a better perception of well-being (albeit only the Jonckheere–Terpstra test is significant in this “typology 1 vs. 3” comparison).

As a final analysis, Table 5 reports the quantile regression results for the quartiles of the perceived well-being indicator (Figure 3A), in particular, the standardized parameter estimates, which provide the (standardized) effect size that each predictor has on the perceived well-being indicator. For the same quartile, the quantile regression was built in two different versions, one unadjusted, including only the dummy variables of the lifestyle typologies (excluding the reference typology 1, i.e., SedSlee-QunPha NEG (Figure 2)), and the other adjusted, with the further insertion of sex, age, and their interaction.

Several remarks are worth making. First, the variations observed on the $R^{1}$ index are minimal overall, both in the comparisons between unadjusted and adjusted models with the same quartile fixed, and in the comparisons across different quartile equations. Nonetheless, the third quartile seems to have a slightly better explanation of its variation than the other two quartiles, both in the unadjusted ($R^{1}\left(0.75\right)=0.060$) and adjusted ($R^{1}\left(0.75\right)=0.084$) models. Moreover, in this latter case, a stronger contribution by the control variables, sex and age, appears. Second, in all the models, controlling for or not for sex and age, the effect sizes of the lifestyle typologies appear in the same ranking, though they do not differ significantly from the effect of the reference typology 1 in all the equations. In any given equation, typology 2 (SedSlee NEG-QunPha POS) presents effect sizes of smaller magnitude on the perceived well-being indicator than those of typology 3 (SedSlee POS-QunPha NEG), and in turn, typology 3 has effect sizes of smaller magnitude than those of typology 4 (SedSlee-QunPha POS). This result further indicates the crucial, positive role of spending less sedentary time and sleeping more hours at night on the children’s and teens’ perceived well-being.

Third, the effects of typologies 3 and 4 differ significantly from those of typology 1 in the equations of the first (τ=0.25) and third (τ=0.75) quartiles of the perceived well-being indicator, both in the unadjusted and adjusted models, while no significant result is detected in the equations of the second quartile (τ=0.5). Moreover, for both typologies 3 and 4, a sort of quadratic trend can be noted when moving from the first quartile equation through the second quartile equation to the third quartile equation. Specifically, regarding the unadjusted models, the effect sizes of typology 3 on the first quartile (${\hat{\eta }}_{3}^{\left(0.25\right)}=0.723$, *p* = 0.017) and the third quartile (${\hat{\eta }}_{3}^{\left(0.75\right)}=0.682$, *p* = 0.013) are both estimated to be greater than its effect size on the second quartile (${\hat{\eta }}_{3}^{\left(0.5\right)}=0.458$, *p* = 0.056). Similarly, the effect sizes of typology 4 on the first quartile (${\hat{\eta }}_{4}^{\left(0.25\right)}=1.091$, *p* = 0.002) and the third quartile (${\hat{\eta }}_{4}^{\left(0.75\right)}=0.955$, *p* < 0.001) are both estimated to be greater than its effect size on the second quartile (${\hat{\eta }}_{4}^{\left(0.5\right)}=0.484$, *p* = 0.053). Analogous remarks can be made for the adjusted models. These findings then suggest that the effects of typologies 3 and 4 may be stronger on children and teens with the lowest and highest levels of well-being perceptions. For instance, considering the adjusted models, i.e., net of sex and age effects, a typology change from 1 to 3 (i.e., spending less sedentary time and sleeping more hours at night) for the 25% of children and teens with worse perceptions of their well-being could help raise their perceived well-being indicator score by 0.672 (95% CI: (0.036, 1.308), *p* = 0.038); a typology change from 1 to 3 for the 25% of children and teens with better perceptions of their well-being could help raise their perceived well-being indicator score by 0.595 (95% CI: (0.061, 1.130), *p* = 0.029).

Fourth, the effects of sex, age, and their interaction are not significant in all the equations, except the age effect for the third quartile. In this case, the estimate is ${\hat{\beta }}^{\left(0.75\right)}=-0.264$, (95% CI: (−0.487, −0.041), *p* = 0.020), and it denotes the impact of age in the female reference group on the third quartile of the perceived well-being indicator, regardless of the lifestyle typology membership. This result indicates that a one-year increase in age in the female group leads to a decrease of 0.264 in the third quartile of the perceived well-being indicator, regardless of the lifestyle typology. Moreover, since the sex-by-age interaction is not significant (${\hat{\gamma }}^{\left(0.75\right)}=0.071$, *p* = 0.721), there is no significant difference in the age effect between females and males (the age effect for males is computed as: ${\hat{\beta }}^{\left(0.75\right)}+{\hat{\gamma }}^{\left(0.75\right)}=-0.193$). Therefore, overall, the variation in the quartiles of the perceived well-being indicator seems not to be affected by sex and age, but by children’s and teens’ lifestyle typology memberships. The only exception regards children and teens with higher levels of perceived well-being, where increasing age seems to play a worsening role in their well-being perceptions.

Table 6 reports the results of the quantile regression analysis performed for the quartiles of the BMI indicator (Figure 3C). Although briefly, a few remarks are worth making. First, unlike Table 5, the observed increments in the $R^{1}$ index values are now more pronounced when moving from unadjusted to adjusted models with the same quartile fixed. That is symptomatic of a stronger contribution by the control variables, sex and age, in explaining the quartile variations in the BMI indicator. Once again, the third quartile seems to have a slightly better explanation of its variation than the other two quartiles, both in the unadjusted ($R^{1}\left(0.75\right)=0.049$) and adjusted ($R^{1}\left(0.75\right)=0.157$) models. Second, with the only exception of the adjusted model for the second quartile, typologies 2, 3, and 4 have negative effects on the BMI indicator, i.e., they seem to contribute to reducing the BMI indicator scores compared to the reference typology 1, especially typology 4 in the unadjusted equations for the second and third quartiles. However, the typology effects are not significant in the adjusted models, so there is not enough empirical evidence to conclude that the considered lifestyle typologies could contribute to reducing the BMI indicator scores effectively. Third, in the adjusted models, the only significant effect is that of age, which, as expected, is positive in all three quartile equations. Conversely, no significant difference is detected between females and males in the quartiles of the BMI indicator.

## 4. Discussion

This study shows that lifestyle worsens with age and influences overall well-being perception in children and teens. Notably, an advanced statistical approach based on the nonlinear principal component analysis [15,25] allowed us to unveil four different lifestyle typologies, defined considering the combination of the two main indicators reflecting, respectively, the role of sedentary behavior and sleep hours (SedSlee indicator) and the quality of nutrition and physical activity volume (QunPha indicator). The healthier the lifestyle, the better the overall well-being perception is (represented by the indicator built considering the individual perceived quality of health, sleep, and academic performance).

CNCD prevention represents one main goal of public health. The initial phases of life are the best periods to teach and enforce healthy behaviors [1], which must be maintained for life. Schools and families play a pivotal role in offering educational and intervention programs to promote health in children and teens, and the intervention may be more effective if the utilized strategy is tailored to children’s and teens’ characteristics and needs.

The classical approach to CNCD prevention [40,41], which considers assessing cardiometabolic risk factors, such as arterial pressure level, lipid profile, BMI, smoking, and plasma glucose levels, may not always be applicable and effective in the healthy young population for ethical, economic, and organizational reasons. This approach may be more useful in adults when the goal is to determine the risk of developing a major cardiovascular disease in the next ten years or to tailor specific drug treatments. Conversely, when the goal is to discover, in the young population, elements that may drive toward the possibility of developing CNCD in the long term (more than ten years), a different approach may be more suitable [42]. To this end, focusing on lifestyle may be more appropriate. An unhealthy lifestyle is generally present before the occurrence of conventional risk factors and may even be present at young ages, particularly in teens [43] and young adults [44]. Moreover, the absence of traditional cardiometabolic risk factors (such as obesity or dyslipidemia) may mislead young individual and even their parents towards the idea that no change is required to foster their own or their children’s future health. Conversely, considering the association between a healthy lifestyle and well-being [2], improving children’s present well-being and performance [45] might represent an important goal that merits being pursued, favoring a proactive role toward behavioral changes.

In this observational study, we assessed the lifestyle and well-being of a group of children/teens at school to obtain data helpful for tailoring a possible educational strategy to the characteristics and needs of the studied group. The questionnaire employed mainly focuses on nutrition (AHA score), exercise (performed METs·minutes/week), sedentary behavior (hours/week), and sleep (hours/night), combining the need to have essential information to build a few indicators using advanced statistical methods with the need to assess main children’s lifestyle features in a simple way. A similar questionnaire filled in by an adult employee population clearly showed that young adults (age < 30 years) were characterized by the worst lifestyle before the occurrence of conventional risk factors [44] as compared to older subjects and that employees meeting current physical activity recommendations (threshold = 600 METs·minutes/week) were characterized by a better psychological and nutrition profile, and a better perception of job performance, sleep, and health quality [46].

We found that our study population meets the WHO physical activity guidelines (Table 1 and Table 2) and that BMI percentiles were perfectly in the normal range in all considered age classes, suggesting that physical inactivity and overweight were of no concern in this population. However, we observed that the AHA diet score—an index of nutrition quality (and not of caloric intake)—was lower than desired (a score of 4–5 is considered “ideal health” [19]) in most children/teens and that this index worsened as age increases. This result suggests that focusing on nutrition quality (preferring fruit/vegetable, fish, whole grain carbohydrates, and reducing salt-rich food and drinks containing sugar) may be worthwhile in this specific, nonoverweight population. Notably, sedentary time increased, while sleep hours and the perception of sleep quality decreased as age increased, suggesting that these lifestyle components deserve improvement. Therefore, a tailored strategy to prevent CNCD and ameliorate health in this specific population might address the role of sedentary behavior, sleep, and nutrition quality rather than only promoting sports and fighting overweight, as is usually carried out in many (not tailored) educational campaigns. Emphasizing only these latter lifestyle changes could result in no motivation to change one’s lifestyle. Vice versa, evidencing the presence of behaviors linked to poor health might foster a proactive role toward a healthy change, particularly in parents.

An advanced statistical approach also permitted the observation that lifestyle influences overall well-being perception in children and teens. Two lifestyle indicators emerged from the analysis, from which four different typologies of children/teens characterized by different lifestyles were derived. Of interest is that structured exercise and sedentary behavior did not cluster into the same indicator (Table 3 and Figure 2), suggesting that the obtained finding fits with the concept that sedentary behavior [8] is not synonymous with physical inactivity [9]. Instead, it means that if a child meets physical activity recommendations but spends many hours in sedentary behavior, the latter may reduce the beneficial effects of exercise [10]. The impact of sedentary behavior on health is strong, and previous research found a statistically significantly higher risk of death for sedentary times of 9.5 or more hours daily [47]. Unfortunately, physical activity declines and sedentary behavior increases during adolescence.

On the other hand, reducing sedentary behavior is associated with a reduction in CNCD. As stated before, these observations must be considered when planning prevention programs and education for youth. Usually, the attention is directed towards fostering participation in structured exercise programs, to be part of sports teams, etc. This strategy, albeit fundamental, might be accompanied by a parallel intervention to reduce sedentary behaviors, particularly in those children/teens who encounter major barriers in participating in sports, such as lack of skills, motivation, enjoyment, and peer support, as well as feeling shy about physical activity in public. Reduction in sedentary behavior is part of the WHO recommendations [7], and it is related not only to the reduction in screen media use but also to the management of other social and behavioral factors, such as the lack of peers to play with or the habits of using elevators and transportation even for short distances.

The concept of children’s and teens’ well-being is attracting more and more attention. Declines in well-being have severe implications for communities and individuals [45]. Many studies and systematic reviews revealed the association between children’s subjective well-being and performance in their future life and behavior [45]. Well-being is considered a complex and multidimensional construct that embodies many factors [6], including physical, mental, and psychological factors. There is a general agreement in indicating that the quality of life [6], perception of health [48], and perception of academic performance [6] are among the significant domains children and youth consider as value components of their well-being, and they are frequently considered also in adult well-being assessment [49]. In this study, we considered three simple questions (related to the perceived quality of health, sleep, and academic performance) to inquire about well-being aspects, fully aware that this approach may be simplistic and many other factors such as socioeconomic status, perception of illness, happiness, feeling good and/or safe, perception of fun, stress, etc., should have been assessed [3,48]. However, long and complex questionnaires may limit their reliability [50,51], especially if proposed to children and their parents in a clinical setting, even outside research protocols. Moreover, we considered that balancing the number of questions devoted to inquiry on different aspects (exercise, sedentary behavior, nutrition, well-being, etc.) was important. The employed lifestyle questionnaire is easy to use, not time-consuming (only 20 questions), and addresses the main lifestyle behaviors (see Section 2.2) and three main aspects determining children’s well-being.

The nonlinear principal component analysis [15] permitted us to define a single well-being perception indicator (Table 4) and to observe that lifestyle significantly influences overall well-being perception in children and teens (Figure 3A,B and Table 5). The healthier the lifestyle, the better the overall well-being perception is (Figure 3A,B and Table 5). The role of lifestyle in influencing children’s and teens’ well-being is well-known in the literature [3,5,6,11]. In this study, we showed, in a simple, cost-effective way, that two uncorrelated dimensions (represented, respectively, by sedentary behavior and sleep (SedSlee indicator) and quality of nutrition and physical activity (QunPha indicator)) might significantly impact well-being. The combination of these two indicators allowed four different lifestyle typologies to be derived, with typology 1 being the worst and typology 4 the healthiest (Figure 2). Our analyses showed that these typologies were characterized by different well-being perceptions, which are better in healthier groups. This result was found even after adjusting for sex and age, suggesting that the relationship between lifestyle and well-being perception is not spurious or determined by these important parameters (Figure 3B and Table 5).

On the contrary, the relationship between BMI and lifestyle was no longer observed after adjusting for age and sex, as expected (Figure 3C,D and Table 6). We decided to involve BMI in the analyses but not in percentiles, as instead recommended by the guidelines [52], for one main reason: since the results obtained for BMI and the BMI indicator were largely expected, this gave us a sort of heuristic validation of the methodology used to build the various indicators, define the lifestyle typologies, and analyze the perceived well-being across typologies also controlling for sex and age effects. In particular, the fact that the sex-by-age-adjusted BMI indicator distribution was no more significantly different across the lifestyle typologies (Figure 3D) made the findings achieved for the perceived well-being indicator more reliable (Figure 3B).

In this study, we took the opportunity offered by a routine clinical assessment (mandatory by law in our country) to assess lifestyle and well-being in a simple manner, having in mind the goal to go beyond the simple release of the clearance for noncompetitive sports participation and to get data useful to tailor intervention to foster health and well-being. This approach is sustainable both from an organizational and economic point of view, with obvious practical advantages. Its novelty is related to translating into a practical approach “the change in point of view”, which characterizes modern scientific literature [42,53,54], adding health behaviors (exercise, nutrition, sleep, etc.), derived from lifestyle assessment, to health factors (blood lipids, blood pressure, glucose levels, etc.), derived from usual clinical assessment, to define cardiometabolic health. Moreover, it underlines the important issue of fostering well-being in youth, which represents an urgent matter in modern society, furnishing an example of a practical strategy.

### Limitations

The study presents some limitations. First, the role of arterial pressure (measured 2–3 times) deserves a comment. We observed that SAP and DAP percentiles were higher in children than teens, albeit in normal ranges. This condition might be explained considering that the youngest subjects were more worried and anxious because of the medical assessment than the oldest ones, who were instead used to undergoing it to obtain clearance to perform sports. Stress is a well-known, powerful condition capable of activating the autonomic nervous system with a consequent acute increase in arterial pressure [55]. In our population, the encounter with a physician and the ECG execution might have represented stressors, particularly in the youngest subjects. This critical bias drove us to refrain from involving systolic and diastolic arterial pressure in constructing statistical indicators and analyzing them across lifestyle typologies. Second, self-reports about lifestyles present some issues that may limit the reliability of the results due to various forms of response biases, which are common to all self-reported data. For instance, some participants may have a tendency always to choose the midpoint or extremes of a response scale. Others might answer in a way that puts them in a positive light, according to what they perceive as acceptable by social norms. Others may not fully understand the questions, or use different definitions for terms, or do not have an accurate perception of their internal states. All these aspects could create spurious associations if the response tendency is linked to other variables, or create associations that do not reflect the “true” causal relationships, making it complicated to draw causal inference. Nevertheless, this is a well-known problem for any questionnaire. In this study, we did our best to minimize this issue, presenting the questionnaire to the families with a letter that explained the importance of the questionnaire close to the medical assessment, and specifically asking parents to fill it in, discussing every question with their sons/daughters. Moreover, the specific study design of this research (observational) did not allow us to infer causation. Third, these findings are specific to the studied population and may not apply to populations with far different characteristics because the subjects voluntarily filled in the questionnaire, and no probabilistic sampling mechanism was used. In addition, such findings are specifically intended to be helpful for selected institutions so that they can propose strategies to improve their students’ lifestyles and foster well-being tailored to their specific characteristics. Fourth, due to privacy reasons (albeit in our country), it was not possible to render such a lifestyle assessment mandatory for all students of the considered school complex. In addition, we had to exclude from the statistical analysis the questionnaires filled in by children/parents who did not give us consent to use them for research purposes, but only for clinical reasons (information necessary to release the clearance for sports activities). Finally, we did not assess other potential confounders (socioeconomic status, parental influence, screen time specifics, etc.) or other factors potentially impacting the well-being to limit the time required to fill in the questionnaire and then improve subjects’ compliance and questionnaire reliability.

## 5. Conclusions

This study showed that a simple assessment of lifestyle and well-being using a short questionnaire, concomitantly with a routine clinical assessment, may furnish important information potentially valuable for tailoring preventive strategies to foster health and well-being in the present and prevent CNCD in the future in the considered population. In particular, the possibility of unveiling specific groups that deserve to improve specific lifestyle components (in this case, represented by poor nutrition quality, sedentary behavior, and insufficient sleep hours) might be more effective than simply counseling to increase participation in sports activities and avoid overweight and obesity. From a practical point of view, a campaign aiming to improve the well-being and health of the group of children who filled out the questionnaire might benefit from addressing the positive effects of reducing sedentary time, improving sleep and quality of nutrition, without stressing only the importance of participating in sports and reducing overweight/obesity, conditions that were not an issue in this population. These data might be useful in designing future research on a vast population of students, considering different sociocultural backgrounds, to define a model for assessing lifestyle and well-being that might be added to routine clinical evaluation, whose derived data might be capable of tailoring intervention to foster health and well-being.

## Figures and Tables

**Table 1:** Summary statistics (median ± MAD) of the studied variables within sex and over the whole subject set, and two-tailed Kruskal–Wallis and median MC significance tests ^1^.

Variables	Female	Male	All
Age (years)	10.25 ± 2.50	10.08 ± 2.17	10.25 ± 2.33
Anthropometrics and hemodynamics			
Weight (kg)	32.30 ± 7.60	35.25 ± 9.60	34.40 ± 8.40
Height (cm)	140.00 ± 12.00	143.75 ± 12.00	143.00 ± 12.50
Waist circumference (cm) **^,††^	61.00 ± 5.00	65.00 ± 6.00	63.00 ± 6.00
Body Mass Index—BMI (kg/m^2^) ^†^	16.18 ± 1.79	17.05 ± 1.67	16.60 ± 1.68
BMI percentiles *	43.00 ± 27.00	55.50 ± 24.50	48.00 ± 27.00
Systolic arterial pressure—SAP (mmHg) **^,††^	100.00 ± 10.00	110.00 ± 7.50	105.00 ± 5.00
Diastolic arterial pressure—DAP (mmHg)	60.00 ± 5.00	65.00 ± 5.00	62.00 ± 3.00
SAP percentiles *	61.00 ± 25.00	74.50 ± 17.50	65.00 ± 23.00
DAP percentiles *	53.00 ± 18.00	65.00 ± 20.00	59.00 ± 18.00
Lifestyle variables			
Hours/week of sedentary behavior	44.00 ± 9.00	47.50 ± 10.50	46.00 ± 9.60
Hours/night of sleep	9.00 ± 1.00	9.00 ± 0.25	9.00 ± 0.50
Volume of moderate and vigorous physical activity (METs, minutes/week) **^,†^	1680.00 ± 480.00	2120.00 ± 760.00	1840.00 ± 720.00
American Heart Association Healthy Diet Score	2.00 ± 1.00	2.00 ± 1.00	2.00 ± 1.00
Perceived quality scales			
Sleep	9.00 ± 1.00	9.00 ± 1.00	9.00 ± 1.00
Academic performance	8.00 ± 1.00	8.00 ± 1.00	8.00 ± 1.00
Health	9.00 ± 1.00	9.00 ± 1.00	9.00 ± 1.00

^1^ The Monte Carlo (MC) tests were based on 10,000 samples. Two-tailed Kruskal–Wallis test: $H_{0}:\tau_{1}=\tau_{2}$ against $H_{1}:\tau_{1}\neq \tau_{2}$, where $\tau_{1}$ is the female effect and $\tau_{2}$ the male effect on variable *X*. Significance level: * *p* < 0.05, ** *p* < 0.01. Two-tailed median test: $H_{0}:\theta_{1}=\theta_{2}$ against $H_{1}:\theta_{1}\neq \theta_{2}$, where $\theta_{1}$ is the median of *X* in females and $\theta_{2}$ the median of *X* in males. Significance level: ^†^ *p* < 0.05, ^††^ *p* < 0.01.

**Table 2:** Summary statistics (median ± MAD) of the studied variables within age classes and two-tailed Kruskal–Wallis and median MC significance tests ^1^.

Variables	5–7 Years	8–10 Years	11–12 Years	13–14 Years
Anthropometrics and hemodynamics				
Weight (kg) ***^,†††^	24.20 ± 2.30	30.85 ± 4.20	41.95 ± 4.95	50.55 ± 4.95
Height (cm) ***^,†††^	125.00 ± 4.00	137.75 ± 5.75	151.50 ± 4.00	166.00 ± 5.25
Waist circumference (cm) ***^,†††^	57.00 ± 3.00	61.00 ± 5.00	66.50 ± 3.50	70.00 ± 4.50
Body Mass Index—BMI (kg/m^2^) ***^,†††^	15.32 ± 0.86	16.41 ± 1.43	17.97 ± 1.66	18.77 ± 1.90
BMI percentiles	43.00 ± 22.00	46.50 ± 23.00	54.50 ± 30.50	53.50 ± 28.50
Systolic arterial pressure—SAP (mmHg) ***^,†††^	100.00 ± 5.00	110.00 ± 5.00	105.00 ± 10.00	110.00 ± 10.00
Diastolic arterial pressure—DAP (mmHg)	62.00 ± 3.00	65.00 ± 5.00	60.00 ± 1.50	60.00 ± 2.50
SAP percentiles ***^,†††^	67.00 ± 12.00	83.50 ± 8.50	63.50 ± 29.50	39.50 ± 21.00
DAP percentiles ***^,†††^	71.00 ± 15.00	68.00 ± 18.00	45.00 ± 9.50	39.50 ± 13.50
Lifestyle variables				
Hours/week of sedentary behavior ***^,†††^	38.50 ± 7.00	47.00 ± 6.45	52.25 ± 12.50	54.25 ± 10.75
Hours/night of sleep ***^,†††^	9.00 ± 1.00	9.00 ± 0.00	8.50 ± 0.50	8.00 ± 0.00
Volume of moderate and vigorous physical activity (METs, minutes/week)	1920.00 ± 720.00	1740.00 ± 660.00	1780.00 ± 820.00	1920.00 ± 720.00
American Heart Association Healthy Diet Score *	3.00 ± 1.00	2.00 ± 1.00	2.00 ± 1.00	2.00 ± 1.00
Perceived quality scales				
Sleep *	9.00 ± 1.00	9.00 ± 1.00	9.00 ± 1.00	8.50 ± 0.50
Academic performance ***^,†††^	9.00 ± 1.00	9.00 ± 1.00	8.00 ± 1.00	8.00 ± 1.00
Health	9.00 ± 1.00	9.00 ± 1.00	9.00 ± 1.00	9.00 ± 1.00

^1^ The Monte Carlo (MC) tests were based on 10,000 samples. Two-tailed Kruskal–Wallis test: $H_{0}:\tau_{1}=\tau_{2}=\tau_{3}=\tau_{4}$ against $H_{1}:\tau_{j}\neq \tau_{v}$ for at least one j≠v, where $\tau_{j}$ is the effect of the *j*-th age class on variable *X*. Significance level: * *p* < 0.05, *** *p* < 0.001. Two-tailed median test: $H_{0}:\theta_{1}=\theta_{2}=\theta_{3}=\theta_{4}$ against $H_{1}:\theta_{j}\neq \theta_{v}$ for at least one j≠v, where $\theta_{j}$ is the median of variable *X* in the *j*-th age class. Significance level: ^†††^ *p* < 0.001.

**Table 3:** PRINCALS applied to the four lifestyle variables arrested to the first two dimensions: Component loadings rotated with the varimax method.

	Dimension 1			Dimension 2		
Variables	Loadings ^1^	90% Bootstrap CIs ^2^		Loadings ^1^	90% Bootstrap CIs ^2^	
Hours/week of sedentary time	−0.773 •	−0.844	−0.656	0.292	0.010	0.501
Hours/night of sleep	0.752 •	0.647	0.826	0.351	0.161	0.489
AHA score	0.224	−0.039	0.519	0.740 •	0.384	0.840
METs of moderate and vigorous physical activity	−0.201	−0.381	0.216	0.678 •	0.417	0.860
Variance Accounted For—VAF (%)	32.63%	31.43%	37.69%	29.12%	25.53%	31.34%
Cumulative VAF (%)	32.63%	31.43%	37.69%	61.75%	59.46%	66.33%

^1^ Black circles mark the loadings greater than, or equal to, 0.6 in absolute value. Dimension 1 = Indicator of sedentary behavior and sleep (acronym: SedSlee); Dimension 2 = Indicator of quality of nutrition and physical activity volume (acronym: QunPha). ^2^ CIs = confidence intervals based on 5000 balanced and stratified bootstrap samples.

**Table 4:** PRINCALS applied to the three perceived quality scales and BMI arrested to the first two dimensions: Component loadings rotated with the varimax method.

	Dimension 1			Dimension 2		
Variables	Loadings ^1^	90% Bootstrap CIs ^2^		Loadings ^1^	90% Bootstrap CIs ^2^	
Perceived health quality	0.808 ▲	0.716	0.849	0.103	−0.169	0.342
Perceived academic performance quality	0.756 ▲	0.629	0.806	−0.151	−0.390	0.030
Perceived sleep quality	0.748 ▲	0.572	0.813	−0.193	−0.497	0.033
BMI	−0.087	−0.225	−0.019	0.981 ▲	0.953	0.999
Variance Accounted For—VAF (%)	46.82%	43.25%	51.94%	23.79%	22.08%	26.76%
Cumulative VAF (%)	46.82%	43.25%	51.94%	70.62%	68.41%	75.32%

^1^ Black triangles mark the loadings greater than, or equal to, 0.6 in absolute value. Dimension 1 = Indicator of perceived well-being; Dimension 2 = Indicator of BMI. ^2^ CIs = confidence intervals based on 5000 balanced and stratified bootstrap samples.

**Table 5:** Quantile regressions for the quartiles of the perceived well-being indicator: Standardized parameter estimates, 95% bootstrap CIs, and significance *t*-tests concerning the models including the lifestyle typologies as predictors, also controlling for sex and age effects ^1^.

**1st Quartile (** τ=0.25 **)**								
	**No adjustment for sex and age** $R^{1}\left(0.25\right)=0.044$				**Adjusting for sex and age** $R^{1}\left(0.25\right)=0.057$			
**Variables**	**Stand.est.**	**95% CIs**	***t* Value**	***p*-Value**	**Stand.est.**	**95% CIs**	***t* Value**	***p*-Value**
Intercept	−1.225	(−1.721, −0.729)	−4.867	**<0.001**	−1.013	(−1.516, −0.509)	−1.519	**<0.001**
Typology 2	0.528	(−0.160, 1.215)	1.513	0.132	0.572	(−0.107, 1.251)	1.652	0.098
Typology 3	0.723	(0.129, 1.317)	2.400	**0.017**	0.672	(0.036, 1.308)	2.077	**0.038**
Typology 4	1.091	(0.397, 1.785)	3.096	**0.002**	0.902	(0.231, 1.572)	2.645	**0.009**
sex					−0.193	(−0.666, 0.280)	0.974	0.423
age					0.014	(−0.264, 0.292)	0.100	0.920
sex-by-age					−0.252	(−0.650, 0.145)	−1.250	0.213
**2nd Quartile (** τ=0.5 **)**								
	**No adjustment for sex and age** $R^{1}\left(0.5\right)=0.041$				**Adjusting for sex and age** $R^{1}\left(0.5\right)=0.045$			
**Variables**	**Stand.est.**	**95% CIs**	***t* Value**	***p*-Value**	**Stand.est.**	**95% CIs**	***t* Value**	***p*-Value**
Intercept	−0.200	(−0.591, 0.190)	−1.012	0.313	−0.188	(−0.581, 0.205)	−0.941	0.348
Typology 2	0.135	(−0.285, 0.556)	0.634	0.527	0.146	(−0.399, 0.691)	0.528	0.598
Typology 3	0.458	(−0.011, 0.927)	1.925	0.056	0.455	(−0.049, 0.959)	1.779	0.077
Typology 4	0.484	(−0.007, 0.975)	1.942	0.053	0.475	(−0.103, 1.052)	1.620	0.107
sex					−0.031	(−0.442, 0.380)	−0.150	0.881
age					0.008	(−0.256, 0.273)	0.063	0.950
sex-by-age					−0.135	(−0.537, 0.266)	−0.665	0.507
**3rd Quartile (** τ=0.75 **)**								
	**No adjustment for sex and age** $R^{1}\left(0.75\right)=0.060$				**Adjusting for sex and age** $R^{1}\left(0.75\right)=$ 0.084			
**Variables**	**Stand.est.**	**95% CIs**	***t* Value**	***p*-Value**	**Stand.est.**	**95% CIs**	***t* Value**	***p*-Value**
Intercept	0.208	(−0.166, 0.583)	1.095	0.275	0.353	(−0.174, 0.880)	1.320	0.188
Typology 2	0.261	(−0.264, 0.785)	0.980	0.328	0.250	(−0.315, 0.815)	0.871	0.385
Typology 3	0.682	(0.147, 1.216)	2.513	**0.013**	0.595	(0.061, 1.130)	2.194	**0.029**
Typology 4	0.955	(0.401, 1.509)	3.399	**<0.001**	0.718	(0.025, 1.411)	2.043	**0.042**
sex					−0.176	(−0.511, 0.159)	−1.036	0.301
age					−0.264	(−0.487, −0.041)	−2.336	**0.020**
sex-by-age					0.071	(−0.321, 0.464)	0.358	0.721

^1^ Reference typology: Typology 1 = SedSlee-QunPha NEG. Legend*:* Stand.est. = Standardized parameter estimates; Typology 2 = SedSlee NEG-QunPha POS; Typology 3 = SedSlee POS-QunPha NEG; Typology 4 = SedSlee-QunPha POS. The *t*-tests are based on the procedure described in [27,28] with standard errors estimated by bootstrap with *B* = 1000 bootstrap samples. Significant *p*-values are written in bold. Typologies 2, 3, 4, and sex are included in the quantile regressions as dummy variables (in the case of sex, with 0 = female and 1 = male); age is included as a continuous variable.

**Table 6:** Quantile regressions for the quartiles of the BMI indicator: Standardized parameter estimates, 95% bootstrap CIs, and significance *t*-tests concerning the models including the lifestyle typologies as predictors, also controlling for sex and age effects ^1^.

**1st Quartile (** τ=0.25 **)**								
	**No adjustment for sex and age** $R^{1}\left(0.25\right)=0.020$				**Adjusting for sex and age** $R^{1}\left(0.25\right)=0.116$			
**Variables**	**Stand.est.**	**95% CIs**	***t* Value**	***p*-Value**	**Stand.est.**	**95% CIs**	***t* Value**	***p*-Value**
Intercept	−0.466	(−0.818, −0.114)	−2.608	**0.010**	−0.572	(−0.853, −0.291)	−4.008	**<0.001**
Typology 2	−0.117	(−0.513, 0.279)	−0.582	0.561	−0.205	(−0.567, 0.157)	−1.119	0.265
Typology 3	−0.237	(−0.621, 0.146)	−1.220	0.224	−0.099	(−0.419, 0.220)	−0.613	0.541
Typology 4	−0.399	(−0.812, 0.015)	−1.899	0.059	−0.119	(−0.475, 0.236)	−0.662	0.509
sex					0.175	(−0.041, 0.390)	1.598	0.112
age					0.339	(0.187, 0.492)	4.395	**<0.001**
sex-by-age					−0.114	(−0.344, 0.116)	−0.975	0.331
**2nd Quartile (** τ=0.5 **)**								
	**No adjustment for sex and age** $R^{1}\left(0.5\right)=0.034$				**Adjusting for sex and age** $R^{1}\left(0.5\right)=0.142$			
**Variables**	**Stand.est.**	**95% CIs**	***t* Value**	***p*-Value**	**Stand.est.**	**95% CIs**	***t* Value**	***p*-Value**
Intercept	−0.041	(−0.323, 0.240)	−0.290	0.772	−0.345	(−0.609, −0.081)	−2.578	0.011
Typology 2	−0.061	(−0.502, 0.381)	−0.270	0.787	0.110	(−0.348, 0.568)	0.474	0.636
Typology 3	−0.302	(−0.641, 0.038)	−1.753	0.081	0.122	(−0.243, 0.486)	0.658	0.511
Typology 4	−0.423	(−0.762, −0.084)	−2.461	**0.015**	0.118	(−0.276, 0.511)	0.589	0.557
sex					0.210	(−0.082, 0.501)	1.416	0.158
age					0.463	(0.279, 0.647)	4.957	**<0.001**
sex-by-age					−0.112	(−0.383, 0.159)	−0.816	0.416
**3rd Quartile (** τ=0.75 **)**								
	**No adjustment for sex and age** $R^{1}\left(0.75\right)=0.049$				**Adjusting for sex and age** $R^{1}\left(0.75\right)=0.157$			
**Variables**	**Stand.est.**	**95% CIs**	***t* Value**	***p*-Value**	**Stand.est.**	**95% CIs**	***t* Value**	***p*-Value**
Intercept	0.843	(0.389, 1.296)	3.664	**<0.001**	0.376	(−0.223, 0.975)	1.236	0.218
Typology 2	−0.281	(−1.045, 0.483)	−0.724	0.470	−0.312	(−0.959, 0.336)	−0.948	0.344
Typology 3	−0.516	(−1.032, −0.001)	−1.974	**0.050**	−0.122	(−0.790, 0.547)	−0.359	0.720
Typology 4	−0.921	(−1.481, −0.362)	−3.245	**0.001**	−0.192	(−0.914, 0.529)	−0.525	0.600
sex					0.218	(−0.165, 0.601)	1.120	0.264
age					0.536	(0.248, 0.824)	3.670	**<0.001**
sex-by-age					0.011	(−0.355, 0.377)	0.059	0.953

^1^ Reference typology: Typology 1 = SedSlee-QunPha NEG. Legend: Stand.est. = Standardized parameter estimates; Typology 2 = SedSlee NEG-QunPha POS; Typology 3 = SedSlee POS-QunPha NEG; Typology 4 = SedSlee-QunPha POS. The *t*-tests are based on the procedure described in [27,28] with standard errors estimated by bootstrap with *B* = 1000 bootstrap samples. Significant *p*-values are written in bold. Typologies 2, 3, 4, and sex are included in the quantile regressions as dummy variables (in the case of sex, with 0 = female and 1 = male); age is included as a continuous variable.

## Data Availability

The dataset used in this study will be uploaded to the Zenodo repository (a link will be provided if the manuscript is accepted). Requests to access the dataset should be directed to daniela.lucini@unimi.it.

## Abbreviations

The following abbreviations are used in this manuscript:

AHA	American Heart Association
BMI	Body Mass Index
CDC	Centers for Disease Control and Prevention
CNCD	Chronic Noncommunicable Diseases
DAP	Diastolic arterial pressure
ECG	Electrocardiogram
FDR	False Discovery Rate
IPAQ	International Physical Activity Questionnaire
IRCCS	Istituto di Ricovero e Cura a Carattere Scientifico (Scientific Institute for Research, Hospitalization and Healthcare)
MAD	Median Absolute Deviation
MC	Monte Carlo
METs	Metabolic Equivalents
METsMV	Moderate and Vigorous Physical Activity Volume Expressed in METs
NEG	Negative
POS	Positive
PRINCALS	Nonlinear (or categorical) Principal Component Analysis
QunPha	Indicator of the Quality of Nutrition and Physical Activity Volume
SAP	Systolic Arterial Pressure
SedSlee	Indicator of Sedentary Behavior and Sleep
VAF	Variance Accounted For

## Appendix A

**Table A1:** Names and descriptions of the variables under study collected over *n* = 225 children and teens.

List of Variables and Their Description	Labels of Variables	Levels of Scaling of the Variables Involved in the PRINCALS Analysis
Sex. 0 = Female; 1 = Male. Sex distribution is in Appendix A Table A2	sex	
Age in years. Distribution of age classes is in Appendix A Table A2	age	
Anthropometric variables		
Weight (in kg)	weight	
Height (in cm)	height	
Waist circumference (in cm)	WC	
Body mass index—BMI (in kg/m^2^)	BMI	metric
BMI in percentiles computed according to the guidelines in [16]	pct.BMI	
Hemodynamic variables	SAP	
Systolic arterial pressure (SAP) by sphygmomanometer (in mmHg)	DAP	
Diastolic arterial pressure (DAP) by sphygmomanometer (in mmHg)	pct.SAP	
SAP in percentiles computed according to the guidelines in [17]	pct.DAP	
DAP in percentiles computed according to the guidelines in [17]	SAP	
Lifestyle variables		
Self-reported number of hours per week of sedentary time. *Percentage distribution with values in intervals:* [9, 36): 23.1%; [36, 50): 35.1%; [50, 65): 29.3%; [65, 154): 12.5%	tot.sedent	metric
Self-reported number of hours per night of sleep. *Percentage distribution with values in intervals:* [6, 8): 5.3%; [8, 9): 27.6%; [9, 10): 48.9%; [10, 11]: 18.2%	sleep.hours	metric
Metabolic Equivalents of moderate and vigorous physical activity per week. *Percentage distribution with values in intervals:* [0, 1200): 19.6%; [1200, 11,760]: 80.4%	METsMV	metric
American Heart Association Healthy Diet Score (in integer scores from 0 to 5). *Percentage distribution:* 0: 2.2%; 1: 18.7%; 2: 35.1%; 3: 36%; 4: 7.6%; 5: 0.4%	AHA.score	Ordinal ^1^—Merged scores: 0–1: 20.9%; 4–5: 8.0%
Perceived quality scales		
Scale of perceived sleep quality (in integer scores from 0 to 10). *Percentage distribution:* 5: 1.8%; 6: 1.3%; 7: 8.0%; 8: 25.8%; 9: 36.0%; 10: 27.1%	qual.sleep	Ordinal ^1^—Merged scores*:* 5–7: 11.1%
Scale of perceived quality of academic performance (in integer scores from 0 to 10). *Percentage distribution:* 5: 1.3%; 6: 6.2%; 7: 15.6%; 8: 34.7%; 9: 26.2%; 10: 16.0%	qual.school	Ordinal ^1^—Merged scores*:* 5–6: 7.5%
Scale of perceived quality of overall health (in integer scores from 0 to 10). *Percentage distribution:* 4: 0.4%; 6: 0.9%; 7: 4.0%; 8: 23.6%; 9: 36.0%; 10: 35.1%	qual.health	Ordinal ^1^—Merged scores: 4–7: 5.3%

^1^ Following the hints in [25], to obtain stable solutions for the PRINCALS dimensions, especially in the bootstrap analysis, we merged scores with marginal frequencies that were too small.

**Table A2:** Cross-table of the 225 participants in the study by sex and age classes ^1^.

			Age Classes (in Years Old)				
			5–7	8–10	11–12	13–14	Total
**Sex**	Female	*N*	25	38	17	27	107
		% within sex	23.4%	35.5%	15.9%	25.2%	100.0%
		% within age class	43.9%	51.4%	37.0%	56.3%	47.6%
	Male	*N*	32	36	29	21	118
		% within sex	27.1%	30.5%	24.6%	17.8%	100.0%
		% within age class	56.1%	48.6%	63.0%	43.8%	52.4%
**Total**		*N*	57	74	46	48	225
		% within age class	25.3%	32.9%	20.4%	21.3%	100.0%
			100.0%	100.0%	100.0%	100.0%	100.0%

^1^ Chi-square MC test: *p* = 0.237 (based on 10,000 sampled tables); Cramer’s V = 0.138, 95% CI: (0.021, 0.221).
